# Differential expression of transcription factors predicted to regulate angiopoietin-1 and -2 genes is associated with specific angiopoietin signatures in RA and PsA synovial tissue

**DOI:** 10.1186/1546-0096-9-S1-P278

**Published:** 2011-09-14

**Authors:** M Frleta, LGM van Baarsen, S Garcia-Perez, D de Launay, M Garrelfs, DM Gerlag, PP Tak, KA Reedquist

**Affiliations:** 1 Division of Clinical Immunology and Rheumatology, Academic Medical Center, University of Amsterdam, Amsterdam, The Netherlands

## 

The angiogenic factors angiopoietin (Ang)-1 and -2 contribute to inflammation and are differentially expressed in synovial tissue of patients with rheumatoid arthritis (RA) and psoriatic arthritis (PsA). The underlying mechanisms for promoting differential expression are unknown. Here, we identified which cells express Ang-1 and Ang-2 in synovial tissue, transcription factors (TFs) predicted to regulate Ang-1 and Ang-2 transcription, and examined expression of these TFs in RA and PsA synovial tissue.

Immunofluorescent double stainings of RA and PsA synovial tissue were performed with CD68, CD163, CD3, CD22 and vWF markers, in combination with antibodies against Ang-1 and Ang-2, using confocal imaging. In silico analysis was conducted to identify TF binding sites in the Ang-1 and Ang-2 promoters, using four different TF binding site prediction programs. RA synovial expression of TFs, Ang-1 and Ang-2 was examined in publicly available gene expression data sets. Candidate TFs were characterized by real-time PCR (qPCR).

Ang-1 production was detected in CD68 and CD163 –positive macrophage, CD3-positive T lymphocytes, and CD22-positive B cells, while Ang-2 expression was restricted to vWF- positive endothelial cells, as well as macrophages. In silico studies identified 34 TFs that were predicted to regulate Ang-1 expression and 22 TFs which are involved in Ang-2 expression and are upregulated in RA synovial tissue (P< 0.05). Expression of these TF was readily detected by qPCR in RA and PsA synovial tissue biopsies.

Our studies suggest that expression of Ang-1 and Ang-2 by different cell types could additional underlie the previously observed specific Ang-1 high signature in RA, compared to PsA. Enhanced expression of Ang-1 and Ang-2 in RA and PsA is strongly associated with expression of specific TFs, and understanding the mechanisms leading to the differential expression of these TFs may give insight into the etiology of RA and PsA.

